# Clinical care ratios for, and tasks undertaken by, allied health professionals: A scoping review of the literature

**DOI:** 10.1371/journal.pone.0312435

**Published:** 2024-11-12

**Authors:** Arabella Brown, Elias El-Achkar, Francesca Fragnito, Sandra Gosnell, Jocelyn Kelly, Daniel Kucharski, Alice Priestly, Saravana Kumar

**Affiliations:** IIMPACT in Health, UniSA Allied Health and Human Performance, University of South Australia, Adelaide, Australia; Endeavour College of Natural Health, AUSTRALIA

## Abstract

**Background:**

With the increasing demand on the global health system and the presence of persistent healthcare disparities, it becomes imperative to utilize the full potential of the healthcare workforce, including allied health professionals (AHPs). To date, no review has mapped the literature on clinical care ratios (CCRs) for AHPs. This information is vital in guiding future work-force planning to address healthcare issues such as understaffed and at capacity hospitals. This scoping review mapped available evidence to offer a comprehensive insight into current AHP CCRs, enabling evidence-based decision-making that ensures optimal care and enhanced system efficiency.

**Methods:**

The scoping review was undertaken using the ‘Preferred Reporting Items for Systematic reviews and Meta-Analyses extension for Scoping Reviews’ (PRISMA-ScR). The search was conducted using Ovid (MEDLINE, Embase, Emcare, PsycINFO), CINAHL, the Cochrane Library, Scopus, PEDro, OTseeker and grey literature. Two reviewers independently screened records, following which data extraction was completed for the included studies. Descriptive synthesis was used to summarize the data.

**Results:**

Of the 7670 records identified, 18 studies were included in this review. Whilst there was variability between AHPs on direct clinical time spent with patients, considerable amount of time was also spent on activities beyond direct clinical care. To offset this, literature highlights opportunities for allied health assistants and students to undertake tasks which would free-up AHPs to undertake more direct and higher-level tasks. However, limitations of findings (variability in measurement, lack of coverage of some professions) limits generalisability.

**Conclusion:**

While the literature highlights considerable variability in CCRs among different AHP disciplines, it does appear nearly a nearly a third of time spent by AHPs are on tasks that are beyond direct clinical care. Future research could explore new workforce models which would free-up AHPs to undertake more direct and higher-level tasks.

## Introduction

The global health care sector is challenged with a shortfall of healthcare workers, estimated to reach ten million by 2023, posing significant challenges to the timely and effective delivery of care [1]. In response to this crisis, a multifaceted strategy is essential, and one particularly promising avenue involves harnessing the expertise of allied health professionals (AHPs). AHPs constitute the second-largest clinical workforce in Australia, closely following the nursing and midwifery sector [2].

Clinical care ratios (CCRs) hold a pivotal role in the realm of healthcare by serving as valuable metrics for quantifying and comparing the clinical and non-clinical workloads of healthcare professionals [3]. Clinical activities involve direct clinical tasks requiring clinician-patient interaction for activities such as assessments, treatments, procedures etc [4]. Clinical activities can also include indirect clinical tasks for a patient but without direct contact with that patient for activities such as administration, documentation, care coordination, discharge planning etc [4]. Beyond workload assessment, CCRs also help improve healthcare delivery by leveraging non-clinical time for activities like developing health promotion materials and public education, contributing to health-supporting environments within communities. Identifying workload discrepancies through CCRs can facilitate efficient resource allocation, reducing the burden of stress and mitigating poor mental health outcomes resulting from inadequate workforce planning [5].

Whilst CRRs have been extensively studied and implemented for nursing and medical staff, the literature offers limited insights into CCRS for AHPs. Research within the nursing field has demonstrated that well-defined CCRs enhance both efficiency and patient care [6]. However, the absence of clear CCRs for AHPs creates barriers to the access of non-clinical time, funding, and resources–essential elements for addressing healthcare system burdens. Consequently, the establishment of public policies to guide CCRs and facilitate workforce planning for AHPs becomes imperative.

Currently, with no universally accepted definition of AHPs, this review adopts an inclusive approach that encompasses all professionals falling within the extensive framework outlined by the Australian Government Department of Health [7] and the Government of South Australia [8]. This comprehensive perspective embraces a wide array of health disciplines, including physiotherapists, podiatrists, social workers, psychologists, occupational therapists, dietitians, speech therapists/pathologists, audiologists, exercise physiologists, and medical radiologists.

With the increasing demand on the global health system and the presence of persistent healthcare disparities, it is essential to fully harness the potential of the healthcare workforce, which includes AHPs. Remarkably, there has been no comprehensive review of the literature on CCRs for AHPs to date. This knowledge is highly important in guiding future workforce planning to address challenges such as understaffed healthcare facilities operating at or above capacity. A thorough understanding of current CCRs will support evidence-based decision-making, ensuring optimal patient care and improved healthcare system efficiency.

The objective of this review was to map the literature on current CCRs and guide workforce planning for AHPs. The primary review question is: ‘what are current CCRs for AHPs working in acute, sub-acute care, outpatient and residential care settings?’ The secondary question is: ‘what are direct and indirect clinical tasks undertaken by AHPs, allied health assistants (AHAs) and students?’ The proposed research question was developed using a population, concept and context (PCC) framework: population (AHPs), concept (CCRs) and context (acute, sub-acute, outpatient and residential care settings).

## Methods

This scoping review was conducted in accordance with the Preferred Reporting Items for Systematic reviews and Meta-Analyses extension for Scoping Reviews (PRISMA-ScR) [9]. A review protocol was developed and published on Open Science Frameworks and can be found at the following link: https://osf.io/ec4bw/.

### Eligibility criteria

Table 1 shows the inclusion and exclusion criteria which were developed using the PCC model for scoping review research questions.

**Table 1:** Inclusion and exclusion criteria.

	Study Characteristics	Population	Concept	Context
**Inclusion**	Quantitative research Qualitative research Systematic reviews	Physiotherapists Podiatrists Social workers Psychologists Occupational therapists Dietitians Speech therapists/pathologists Exercise physiologists Audiologists Medical radiologists Allied health students/interns Allied health assistants	Indirect/direct clinical care ratios	Acute care settings Sub-acute care settings Outpatient settings Residential aged care
**Exclusion**	Conference presentations Opinion articles/commentaries Abstracts Full text unavailable English language unavailable Published duplicates	Pharmacy Medical Nursing Midwifery		Primary care settings

### Study characteristics

Quantitative, qualitative studies and systematic reviews were included. Conference presentations, opinion articles and abstracts were excluded. Published duplicates and non-English publications were excluded. If full text versions for any studies were unable to be sourced, these studies were also excluded.

#### Population

Sources examining AHPs, including physiotherapists, podiatrists, social workers, psychologists, occupational therapists, dietitians, speech therapists/pathologists, exercise physiologists, audiologists, and medical radiation, have been included. Sources relating to allied health students, interns, and assistants were also included. Studies which included medical, nursing, midwifery or pharmacy workforce were excluded.

#### Concept

Studies that reported direct and indirect CCRs were included.

#### Context

Studies with acute, sub-acute, outpatient and residential care settings were included, while those concerned with primary care settings were excluded.

### Information sources

The search strategy aimed to identify commercially published and unpublished grey literature sources. A comprehensive search was conducted of the following databases: Ovid MEDLINE, Ovid Embase, Ovid Emcare, Ovid PsycINFO, CINAHL, the Cochrane Library, Scopus, PEDro and OTseeker from inception to the 3^rd^ of October 2023. These databases were selected due to accessibility and relevance to the research question. The researchers who conducted the database searches, along with the dates the searches were performed, are listed in Appendix 1 in S1 Appendix. To ensure comprehensiveness of the review process and to avoid publication bias, Google and Google Scholar were used to identify grey literature. The first ten pages on these sites were screened to identify relevant articles in a timely manner.

### Search

This review implemented a three-stage search strategy. An initial search was completed on Ovid (Medline and PsycINFO), Scopus, PEDRO, OTseeker, Scopus and CINAHL to identify relevant articles. The abstracts and titles of these articles were examined for recurring keywords and index terms. The second stage was a comprehensive search of all identified data bases with the relevant key terms. Table 2 contains a table of search terms utilized. Finally, pearling was also used to identify any additional articles in the reference lists of eligible research.

**Table 2:** Search terms.

Allied Health Professional		Clinical Care Ratios		Settings
MeSH: Allied Health Personnel/		MeSH: N/A		MeSH: N/A
SYNONYMS OR RELATED TERMS		SYNONYMS OR RELATED TERMS		SYNONYMS OR RELATED TERMS
Physiotherap*	AND	Workload*	AND	Acute*
OR		OR		OR
Podiatr*		Indirect ADJ3 direct		Hospital*
OR		OR		OR
Social work*		Clinical ADJ3 non-clinical		Inpatient*
OR		OR		OR
Psychologist*		Clinical care ratio*		Outpatient*
OR		OR		OR
Occupational therap*		Staffing		Sub-acute*
OR		OR		OR
Dieti?ian*		Workforce ratio*		Healthcare facilit*
OR		OR		OR
Speech therapist*		Staff* ratio*		Rehab*
OR		OR		OR
Exercise therapist*		Workforce plan*		Residential facilit*
OR		OR		OR
Exercise physiologist*		Direct patient car*		Nursing home*
OR		OR		OR
Audiologist*		Indirect patient car*		Aged car*
OR				OR
Psychology staff*				In-patient*
OR				OR
Physical therapist*				Out-patient*
OR				OR
Speech pathologist*				Subacute*
OR				OR
Allied health*				Sub acute
OR				
Medical radiologist*				

The search strategy included truncations, Boolean terms, wildcards, and syntax terms. Boolean terms, ‘AND’ and ‘OR,’ were utilized to combine key terms and narrow the search. Syntax terms, ‘ADJ3,’ were used to limit word proximity. Truncations, use of an asterix (*) at the end of the word, were used to detect sources with different word endings. Wildcards, use of a question mark (?), enabled terms to be found with different spellings. As each database has unique operators, search fields, thesauri (e.g. MeSH, EmTree) and search syntaxes, the terms in Table 2 were adapted accordingly. Search limits for language (English language) was the only limiter used in the search process. Ovid Medline search syntax is presented in Appendix 2 in S1 Appendix.

### Selection of literature

Literature sources, identified by the search strategies, were exported into EndNote X9^®^, a reference management software, to remove duplicates. Remaining articles were imported into Covidence^TM^ Software a screening tool, where a two-stage screening process was conducted. Firstly, each article’s title and abstract were screened by two members of the review team independently against the inclusion criteria to determine eligibility. A third independent reviewer resolved any conflicts. Secondly, full text of identified articles were sourced and each assessed by two members of the review team independently against the inclusion criteria to determine eligibility. A third independent reviewer (SK) resolved any conflicts. If a systematic review was identified for inclusion, to avoiding double-counting of articles, we only included articles that were not included in the systematic review or published after the stipulated search timelines of the systematic review.

### Data items

The data items extracted included the title, author(s), year of publication, country in which research was conducted, study design, sample size and demographics (participant age, gender, occupation, level of training, years of experience), details on CCRs (including calculation method, and nature of clinical and non-clinical tasks), and the relevant setting.

### Data extraction

Relevant data were charted to create a summary of results. A customized data extraction form using Microsoft Excel spreadsheet was created and used to store the extracted data. Piloting of the data extraction form was conducted by the review team. One member of the review team initially extracted all relevant data items using the data extraction spreadsheet, then a second reviewer of the review team reviewed the results to ensure accuracy. Any disagreement or conflicting data was managed via consensus, voting or through a third reviewer (SK). If data was unclear, missing, or required clarification, the reviewers requested additional information from the authors of the article.

### Critical appraisal of individual sources of evidence

As per best practice standards in conducting a scoping review, a formal critical appraisal of the included literature was not undertaken.

### Synthesis of results

A narrative synthesis of the literature was conducted to align with the type of the review and broad focus of the review questions. Summary tables were used to identify CCRs, clinical and non-clinical tasks. The review team was involved in the data synthesis process and supported by the facilitator (SK).

## Results

### Selection of literature

**Fig 1:**
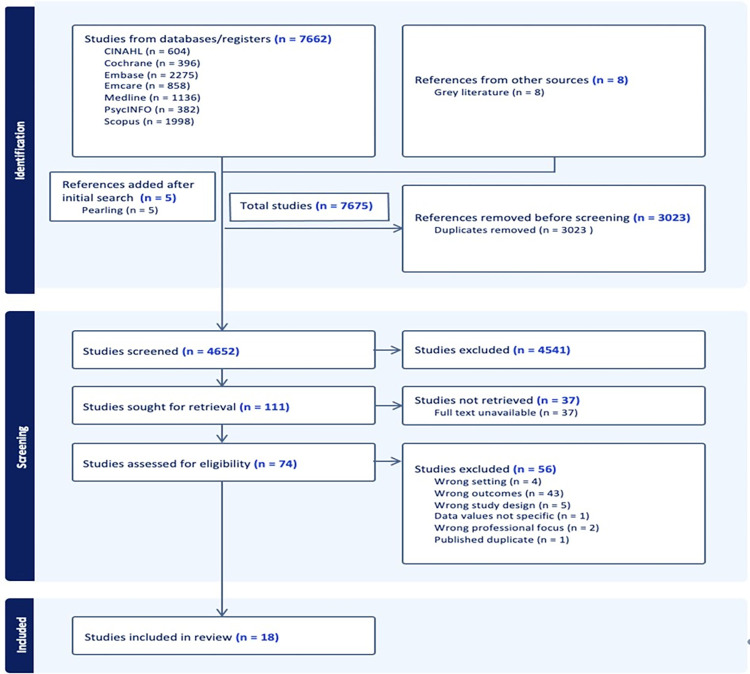
PRISMA flow chart.

As outlined in Fig 1, the initial search identified 7670 potential studies (7662 from primary sources and eight from grey literature). Five additional studies were included after completion of pearling, increasing the total number of studies to 7675. 3023 duplicate studies were removed, leaving 4652 studies for screening. Upon title and abstract screening, 4541 studies did not meet the inclusion criteria. Of the remaining 111 studies, 37 were removed as full text was unavailable, leaving 74 studies for full text screening. 56 studies were excluded for the following reasons: wrong setting (n = 4), wrong outcomes (n = 43), wrong study design (n = 5), data values not specific (n = 1) and wrong professional focus (n = two). One article, Paterson [10], was excluded as a published duplicate as the data was present in the systematic review by Bourne et al. [11]. Therefore, the final number of included studies in this review was 18.

### Characteristics of sources of evidence

#### Study characteristics

Table 3 provides a detailed description of the study characteristics for included studies. All included studies were published between 1983 and 2023. Eight studies explored findings from the United States [12–19]. Six studies included data from Australia [3, 11, 20–23]. Three studies focused on the United Kingdom [11, 24, 25] and two focused on Canada [11, 26]. One study each focused on Ireland [25] and Italy [27]. One study did not specify which country their findings were from [13]. All studies utilised quantitative methods, with one study also incorporating qualitative interviews [22].

**Table 3:** Study characteristics.

Study	Country	Study Design
*Bourne et al*. 2019 [11]	*US*, *Canada*, *Australia*, *UK*	*Systematic review with meta-analysis*
Domenech *et al*. 1983 [13]	Not specified	Quantitative (observational–descriptive (work-sampling))
Dunal *et al*. 2005 [26]	Canada	Quantitative (observational–correlational)
Hall *et al*. 2021 [20]	Australia	Quantitative (experimental–pre-post)
Hand *et al*. 2019 [14]	US	Quantitative (observational–cross-sectional)
Hand *et al*. 2015 [15]	US	Quantitative (observational–descriptive (data collection sheet))
Hearn *et al*. 2017 [3]	Australia	Quantitative (observational–retrospective cohort study)
Iosa *et al*. 2018 [27]	Italy	Quantitative (observational–descriptive (work-sampling technique))
Long *et al*. 2020 [16]	US	Quantitative (observational–descriptive (work sampling))
Meyer and Olsen 1989 [17]	US	Quantitative (observational–descriptive (survey))
Milosavljevic *et al*. 2014 [21]	Australia	Quantitative (observational–prospective cohort study)
Phillips 2015 [18]	US	Quantitative (observational–prospective cohort study)
Pinson *et al*. 2023 [22]	Australia	Explanatory, sequential mixed-methods study design (quantitative; qualitative (case study–interview)
Rudd *et al*. 2009 [24]	UK	Quantitative (observational–descriptive (survey))
Shanklin *et al*. 1988 [12]	US	Quantitative (observational–descriptive (work sampling))
Stachowski and Kondela-Cebulski 1983 [19]	US	Quantitative (observational–descriptive (survey))
Stoikov *et al*. 2021 [23]	Australia	Quantitative (observational–retrospective cohort study)
Ward and O’Riordan 2015 [25]	Ireland, UK	Quantitative (observational–descriptive (survey)); quantitative (observational–descriptive (work-sampling tool))

***Note*:** US = United States of America, UK = United Kingdom

### Population and context

This review assessed the CCRs of AHPs using articles from various settings, as summarised in Table 4. Twelve of the eighteen included studies were in hospital settings [3, 12, 13, 15–18, 20, 21, 23, 25, 27]. Six articles were in varied health care setting such as stroke units, burn care facilities, independent health care organisations, outpatient dialysis clinics and geriatric chronic care centres [11, 14, 19, 22, 24, 26]. The main AHPs that were featured in the included articles were physiotherapists, occupational therapists, dietitians, speech therapists, social workers, medical imaging assistants and AHAs. The AHPs had varying levels of experience ranging from students to clinical supervisors. Across sixteen studies, the CCRs of 4,854 AHP were evaluated, with one study only providing the number of health facilities that were included in their study [24] and one systematic review with varied numbers of health professionals across various studies [11]. Of the 4,854 AHPs, 930 were physiotherapists, 412 were physiotherapy students, 468 were occupational therapists, 2,478 were dietitians, 198 were speech pathologists/therapists, 357 were social workers, four were medical imaging students and seven were AHAs.

**Table 4:** Participant characteristics and context.

Study	Participant Characteristic	Context
Bourne et al. 2019 [11]	*23 studies–qualitative* *Number of studies*: *dietitian (1)*, *OT (4)*, *PT (16)*, *SP (3)* *2–114 clinicians—median of 18*	*Varied public healthcare facilities*
Domenech et al. 1983 [13]	1 x supervisor (1 FTE), 3 x full time PT and 2 x part-time PT, 2 x PT aides, 1 x clerk	General hospital
Dunal et al. 2005 [26]	4 x OT	Geriatric chronic care setting
Hall et al. 2021 [20]	2 x permanent senior CF PT, 2 x junior rotational PT, and 1 x AHA	Adult hospital CF services—inpatient and outpatient
Hand et al. 2019 [14]	14 x dietitians (across 14 dialysis centres)	Out-patient dialysis clinics
Hand et al. 2015 [15]	353 x RDNs (in 78 facilities)	Acute care hospitals
Hearn et al. 2017 [3]	776 x PT, 418 x OT, 198 x SP, 287 x dietitians, 357 x SW	Tertiary hospital
Iosa et al. 2018 [27]	12 x PT, 6 x AHA	Neurorehabilitation hospital
Long et al. 2020 [16]	43 x OT, 50 x PT	Paediatric Hospital
Meyer and Olsen 1989 [17]	283 x dieticians	Hospital
Milosavljevic et al. 2014 [21]	17 x dieticians (across 7 sites) - 11 x senior clinical dietitians, 8 x entry level dietitians	Hospital setting
Phillips 2015 [18]	1,311 x RDNs (across 420 hospitals)	Hospitals
Pinson et al. 2023 [22]	4 x medical imaging assistants (experience ranged <1 year to 8 years (median 4.4 years))	Independent healthcare organisations
Rudd et al. 2009 [24]	PT, OT, ST (across 24 acute stroke units, 33 rehabilitation stroke units, 32 combined stroke units, two neurological rehabilitation units, and one unit refused to declare a type)	Stroke units
Shanklin et al. 1988 [12]	127 x clinical dietitians (across 49 hospitals)	Hospitals
Stachowski and Kondela-Cebulski 1983 [19]	30 x PT, 3 x OT, 1 x unidentified title	Burn care facilities
Stoikov et al. 2021 [23]	412 x PT students, 50 x new graduate PT (across 4 clinical areas in 5 public hospitals)	Public hospitals–inpatient care across musculoskeletal, neurology, cardio-respiratory and orthopaedics
Ward and O’Riordan 2015 [25]	86 x paediatric dietitians (across 45 specialist children’s hospitals; 34 tertiary paediatric units; 7 miscellaneous)	3 x specialist children’s hospitals, 3 x tertiary paediatric units, 2 x community or district hospitals, 1 x specialist children’s hospital

***Note*:** OT = Occupational Therapist, PT = Physiotherapist, AHA = Allied Health Assistant, SP = Speech Pathologist, SW = Social Worker, CF = Cystic Fibrosis, FTE = Full Time Equivalent, RDNs = Registered Dietitian Nutritionists, MSK = musculoskeletal

#### Concept: Clinical care ratio calculation method

Thirty eight percent of the studies utilized practitioner self-recorded methods [12, 15, 18, 22, 23, 25, 26]. Observation accounted for 27% and was employed by Domenech et al. [13]; Hearn et al. [3]; Iosa et al. [27]; Long et al. [16]; and Milosavljevic et al. [21]. Electronic recording methods were utilized in 11% of studies, with Hall et al. [20]employing a Portable Scanning System (Chappell Dean Pty) and Hand et al. [14] utilizing the Work Activity Measurement by Activity Timing (WOMBAT) Software. Additionally, survey questionnaires were used in 11% of studies, with both Meyer and Olsen [17] and Stachowski and Kondela-Cebulski [19] adopting this method. The remaining 11% of studies fell under the ‘other’ category, including Bourne et al. [11] who conducted a meta-analysis of studies and Rudd et al. [24] who employed a survey and statistical analysis. See Table 5 for a summary of the different CCR calculation methods.

**Table 5:** Clinical care ratio calculation method.

Study	Clinical Care Ratio Calculation Method			
Clinical Care Ratio Calculation Method	Observation	Electronic Recording Method (Specify)	Practitioner Self Recorded	Other (Specify)
Bourne et al. 2019 [11]				*Meta-Analysis of Studies*
Domenech et al. 1983 [13]	X			
Dunal et al. 2005 [26]			X	
Hall et al. 2021 [20]		Portable Scanning System (Chappell Dean Pty)		
Hand et al. 2019 [14]		Work Activity Measurement by Activity Timing (WOMBAT) Software		
Hand et al. 2015 [15]			X	
Hearn et al. 2017 [3]	x			
Iosa et al. 2018 [27]	X			
Long et al. 2020 [16]	X			
Meyer and Olsen 1989 [17]				Survey Questionnaire
Milosavljevic et al. 2014 [21]	X			
Phillips 2015 [18]			X	
Pinson et al. 2023 [22]			X	
Rudd et al. 2009 [24]				Survey questionnaire
Shanklin et al. 1988 [12]			X	
Stachowski and Kondela-Cebulski 1983 [19]				Survey Questionnaire
Stoikov et al. 2021 [23]			X	
Ward and O’Riordan 2015 [25]			X	

#### Concept: Clinical care ratios

Within the literature, a number of different CCRs were identified for various allied health professions including physiotherapy, occupational therapy, dietetics, speech pathology, social work and medical imaging assistants (see Table 6). There was no consistency to how CCRs were captured and reported. Whilst some studies reported in a percentage-based data, others quantified CCRs by deducting average time spent on either direct or indirect patient related tasks. Most studies (14 out of 18) utilized a combination of percentages and quantified time spent with patients (minutes) to offer a clear representation of AHP’s clinical time was distributed during their workday. In contrast to this, Bourne et al. [11], Meyer and Olsen [17] and Shanklin et al. [12] chose to express these ratios solely with time spent in minutes or hours on direct or indirect patient related tasks. Notably, 12 studies explicitly calculated both direct and indirect care ratios, differentiating the time allocated to direct patient care and supporting activities.

**Table 6:** Clinical care ratios.

Study	Allied Health Profession	Clinical Care Ratio	
Study	Allied Health Profession	Direct	Indirect
Bourne et al. 2019 [11]	*PT*, *OT*, *D*, *SP*, *student therapists from each profession*.	*>25mins to patients / 60 mins of direct clinical time*	
Domenech et al. 1983 [13]	PT	Direct care 28.45% (46.67 mins of patient related activities)	Indirect care 11.74%
Dunal et al. 2005 [26]	OT	40% direct care	Indirect care 60%
Hall et al.2021 [20]	PT AHA	PT 81% clinical care AHA 90% clinical care	PT non-clinical 19% AHA non-clinical 11%
Hand et al. 2019 [14]	D	Mean amount of direct care time per patient encounter 6.95 +/- 4.05 minutes, min 1.22 mins, max 19.05 mins. 25% direct patient care time	
Hand et al. 2015 [15]	D	Mean time per patient encounter 26.1 +/- 17.5 mins, median 23 mins. 48.4% of each day on individual patient encounters	
Hearn et al. 2017 [3]	PT, OT, D, SP, SW	Entry Level Practitioners: Mean % CCRs PT 83.54%, OT 80.05%, D 73.57%, SP 76.39%, SW 75.63%	
Iosa et al. 2018 [27]	PT	Direct 75.2% = 276 mins	Indirect 5.7% = 21 mins.
Long et al. 2020 [16]	PT, OT	PT-OP 49%, OT-OP 35% direct patient care	OT-IP indirect ranged 32–39%
Meyer and Olsen 1989 [17]	D	Direct 20.4 hours, (42.7-hour week)	Indirect 13.6 hours, 7.3 hours non-patient care (42.7-hour week)
Milosavljevic et al. 2014 [21]	D	IP = 32.1%, OP = 18.3% of time on direct patient care activities	Indirect 23.3% IP and 41.7% OP
Phillips 2015 [18]	D	2.42 patients per hour in direct patient care, 77% of time on direct care activities,	23% of time spent on indirect care activities
Pinson et al. 2023 [22]	Medical Imaging Assistants	Patient related 39%. Direct patient related tasks 23%	Non-patient related tasks 59%, indirect patient related tasks 16%.
Rudd et al. 2009 [24]	PT, OT, SP	PT 40–45 mins, OT 30–45 mins, SP 30–40 mins	
Shanklin et al. 1988 [12]	D	Direct patient care approx 149mins combined.	47% non-direct patient care approx 97.8 mins
Stachowski and Kondela-Cebulski 1983 [19]	PT	Direct patient care 66.9%	Average for indirect = 30.3%.
Stoikov et al. 2021 [23]	PT, Student PT	New grad PT—80% direct patient care, OOS 8.8 per day mean, mean mins 42. Student PT direct patient care 56%, 4.4 OOS	
Ward and O’Riordan 2015 [25]	D	Direct = 58.4% of working day total.	Indirect = 19.2% working day, breaks 6%

***Note*:** PT = Physiotherapist, SP = Speech Pathologist, D = Dietitian, SW = Social Worker, AHA = Allied Health Assistant, OT = Occupational Therapist, OP = Outpatient, OOS = Occasions of Service, IP = In Patient

This scoping review identified the most CCRs for both physiotherapy and dietetics disciplines, each incorporated in nine of the included studies. Physiotherapy had the highest ratio of direct clinical time spent with patients, with an average of 66.3%, ranging from 28.5% [13] to 81% [20]. Bourne et al. [11] illustrated the need for over 25 minutes for every 60 minutes of direct clinical time in physiotherapy. In contrast, Domenech et al. [13] portrays physiotherapist’s allocation of approximately 28.45% for direct patient care and 11.74% for indirect care. Furthermore, Hall et al. [20] revealed that while physiotherapists spend 81% of their time on clinical care, AHAs allocate 90% of their time to this sector. This comparison highlights the importance of having AHAs for physiotherapists, in addition to the direct and indirect tasks within their role that associated with patient care. Stoikov et al. [23] furthers the discussion by exploring the transition from student to new graduate PTs, illustrating how the direct patient care ratio evolves during this progression.

The CCRs in dietetics were also diverse, averaging 52.35% spent in direct clinical activities, with a range from 25% [14] to 77% [18]. Hand et al. [14] reports a mean of 6.95 direct minutes, constituting 25% of a patient encounter, whilst Hand et al. [15] depicts a mean time of 26.1 minutes per patient encounter, with 48.4% of the day devoted to individual patient encounters. Meyer and Olsen [17] add to this complexity by providing a comprehensive view of hours, including 20.4 hours for direct care, 13.6 hours for indirect care, and 7.3 hours for non-patient care in a 42.7-hour workweek. Additionally, Milosavljevic et al. [21] distinguish between inpatient care (IP) and outpatient care (OP), with 32.1% of time allocated to IP and 18.3% to OP. Phillips [18] adds further granularity, indicating that dietitians see approximately 2.42 patients per hour, devoting 77% of their time to direct care activities.

Beyond physiotherapy and dietetics, data from Pinson et al. [22] reveals that medical imaging assistants allocate 39% of their time to patient-related tasks, with 23% dedicated to direct patient-related activities and 16% to indirect patient-related tasks. In the domain of occupational therapy, Dunal et al. [26] offers a CCR of 40% for direct care and 60% for indirect care. While limited in data, Rudd et al. [24] notes the average time spent on direct patient care per patient for occupational therapists.

#### Context: Clinical tasks

All 18 studies defined and/or described the clinical tasks undertaken by AHPs, assistants or students. For clinical tasks, several studies distinguished between direct and indirect clinical care. For the purposes of this scoping review, direct clinical tasks are interpreted as those involving direct therapist-patient interaction [16, 27] with either face-to-face or via email/telephone patient-based activities [16]. In contrast, indirect clinical tasks are tasks undertaken for a specific patient but without direct contact with that patient [27]. The analysis below identifies the direct and indirect clinical tasks categorized by the included studies.

*Direct clinical tasks*. As shown in Table 7 there were nine studies that included physiotherapy; however, five studies did not specify what the direct tasks were comprised of [3, 11, 13, 23, 24], and one study listed tasks but did not categorise them between clinical (direct/indirect) and non-clinical [19]. Treatment was one direct clinical task reported across multiple studies [16, 20, 27]. In terms of occupational therapy, six studies mentioned direct clinical tasks but four [3, 11, 24, 26] did not specify what those tasks included, and one study failed to categorise tasks between clinical and non-clinical time. For dietetics, there were many direct clinical tasks listed across the nine studies. Assessment was the most commonly reported activity, prevalent across five studies [14, 17, 21, 25] with treatment being the next most reported direct activity [14, 17, 21, 25]. For speech pathology and social work, there was no explanation of direct clinical tasks in any of the studies. For AHAs and students, three studies supplied information on direct clinical tasks [20, 22, 23, 27] with treatment, and transfers/transportation being the most commonly reported direct clinical tasks [22, 27].

**Table 7:** Direct clinical tasks undertaken by the relevant AHPs, assistants and students.

Direct-clinical tasks																									
Study	Physiotherapy						OT				Dietetics							Speech Path	Social Work	Allied Health Assistants or Students					
	Treatment	Testing/ Evaluation	MDT Clinics	Transfers/transportation	Communication	Not specified	Patient Scheduling	Treatment	Testing/Evaluation	Not specified	Assessment	Treatment	Testing/Evaluation	Communication	Education	Discharge Planning/Rounds	Not specified	Not specified	Not specified	Assessment	Treatment	Testing/Evaluation	Transfers/transportation	Communication	Not Specified
Bourne et al. 2019 [11]						✓				✓							✓	✓							
Domenech et al. 1983 [13]						✓																			
Dunal et al. 2005 [26]										✓															
Hall et al. 2021 [20]	✓	✓	✓																		✓	✓			
Hand et al. 2019 [14]											✓	✓		✓											
Hand et al. 2015 [15]																	*								
Hearn et al. 2017 [3]						✓				✓							✓	✓	✓						
Iosa et al. 2018 [27]	✓			✓	✓																✓		✓	✓	
Long et al. 2020 [16]	✓	✓			✓		✓	✓	✓																
Meyer and Olsen 1989 [17]											✓	✓	✓												
Milosavljevic et al. 2014 [21]											✓	✓	✓		✓	✓									
Phillips 2015 [18]											✓				✓	✓									
Pinson et al. 2023 [22]																				✓	✓		✓		
Rudd et al. 2009 [24]						✓				✓								✓							
Shanklin et al. 1988 [12]																	*								
Stachowski and Kondela-Cebulski 1983 [19]						*				*															
Stoikov et al. 2021 [23]						✓																			✓
Ward and O’Riordan 2015 [25]											✓	✓		✓		✓									

*Indirect clinical tasks*. There were many indirect clinical tasks listed across the 18 studies, as summarised in Tables 8 and 9. For physiotherapy, the most commonly reported task was documentation which was listed in three studies [16, 20, 27]. However, four studies did not specify what the indirect clinical tasks comprised of [11, 13, 23, 24]. For dietetics, the review authors determined seven broad categories of indirect clinical tasks reported across seven studies. Each reported in five studies [3, 14, 18, 21, 25], the most frequently listed tasks were training/education/research, administration/ management/ planning, communication/meetings/medical rounds each reported in five studies. Three studies [14, 17, 21] each listed documentation and communication as further indirect clinical tasks for dietetics. Six studies regarding occupational therapy mentioned indirect clinical tasks, however, only two of these specifically listed tasks which most commonly reported being administration/management/planning [3, 16]. Only indirect clinical tasks were reported for speech pathology and social work across the studies, with administration/management/planning and training/education/research listed for each discipline in one study [3]. For AHAs and students, administration and documentation were the most commonly reported indirect clinical tasks, each reported in three studies [20, 22, 27], followed by communication reported in two studies [22, 27].

**Table 8:** Indirect clinical tasks undertaken by the relevant allied health professionals, assistants and students.

Indirect-clinical tasks																													
Study	Physiotherapy						Dietetics								OT					Speech Path			Social Work		Allied Health Assistants/Students				
	Administration/Management/Planning	Environmental Set-up/Equipment	Documentation	Training/Education/Research	Communication/Meetings/Medical Rounds	Not specified	Documentation	Communication/Meetings/Medical Rounds	Breaks/Travel	Administration/Management/Planning	Training/Education/Research	ADLs and food service	Supervision	Not specified	Administration/Management/Planning	Documentation	Environmental Set-up	Training/Education/Research	Not specified	Administration/Management/Planning	Training/Education/Research	Not specified	Administration/Management/Planning	Training/Education/Research	Administration	Documentation	Equipment	Communication	Not Specified
Bourne et al. 2019 [11]						✓								✓					✓			✓							
Domenech et al. 1983 [13]						✓																							
Dunal et al. 2005 [26]																			✓										
Hall et al. 2021 [20]	✓	✓	✓																						✓	✓	✓		
Hand et al. 2019 [14]							✓	✓		✓	✓																		
Hand et al. 2015 [15]														*															
Hearn et al. 2017 [3]	✓			✓						✓	✓				✓			✓		✓	✓		✓	✓					
Iosa et al. 2018 [27]	✓		✓		✓																				✓	✓		✓	
Long et al. 2020 [16]		✓	✓		✓										✓	✓	✓												
Meyer and Olsen 1989 [17]							✓	✓		✓																			
Milosavljevic et al. 2014 [21]							✓	✓		✓	✓		✓																
Phillips 2015 [18]								✓		✓	✓	✓																	
Pinson et al. 2023 [22]																									✓	✓		✓	
Rudd et al. 2009 [24]						✓								*					✓			✓							
Shanklin et al. 1988 [12]																													
Stachowski and Kondela-Cebulski 1983 [19]						*													*										
Stoikov et al. 2021 [23]						✓																							✓
Ward and O’Riordan 2015 [25]								✓	✓		✓		✓																

**Table 9:** Non-clinical tasks undertaken by the relevant AHPs, assistants and students.

Non-clinical tasks																																			
Study	Physiotherapy										OT				Dietetics									Speech Path	Social Work	Allied Health Assistants or Students									
	Administration	Breaks	Cleaning	Communication	Documentation	Management/Planning	Meetings	Research	Training/education	Not specified	Breaks	Meetings	Training/education	Not specified	Administration	Breaks	Communication	Meetings	Quality control	Research	Supervision	Training/education	Not specified	Not specified	Not specified	Administration	Breaks	Cleaning/Stock Control/Environment	Communication	Delays	Management	Meetings	Research	Training/education	Not specified
Bourne et al. 2019 [11]										✓				✓									✓	✓											
Domenech et al. 1983 [13]	✓	✓		✓	✓																														
Dunal et al. 2005 [26]														✓																					
Hall et al. 2021 [20]						✓		✓	✓																						✓		✓	✓	
Hand et al. 2019 [14]															✓	✓				✓	✓	✓													
Hand et al. 2015 [15]																							*												
Hearn et al. 2017 [3]										✓				✓									✓	✓	✓										
Iosa et al. 2018 [27]	✓	✓	✓	✓		✓	✓		✓																	✓	✓	✓	✓		✓	✓		✓	
Long et al. 2020 [16]		✓					✓		✓		✓	✓	✓																						
Meyer and Olsen 1989 [17]															✓			✓	✓		✓	✓													
Milosavljevic et al. 2014 [21]															✓		✓					✓													
Phillips 2015 [18]															✓			✓		✓		✓													
Pinson et al. 2023 [22]																										✓		✓		✓		✓			
Rudd et al. 2009 [24]										✓				✓										✓											
Shanklin et al. 1988 [12]																							*												
Stachowski and Kondela-Cebulski 1983 [19]										*				*																					
Stoikov et al. 2021 [23]										✓																									✓
Ward and O’Riordan 2015 [25]																							✓												

*Non-clinical tasks*. Physiotherapy, dietetics, AHAs and students have the greatest number of non-clinical tasks listed across the studies, as seen in Table 9. Three studies listed tasks but did not categorise these, leading the authors to be unable to determine whether they were non-clinical [12, 14, 19]. For physiotherapy, the most commonly listed non-clinical tasks were training/education [16, 20, 27] and breaks [13, 16, 27]. For the six studies that concerned occupational therapy, only three tasks (breaks, meetings, training/education) were specified in one study [16]. For dietetics, administration and training/education were the most common non-clinical tasks, listed in four studies each [14, 17, 18, 21]. For speech pathology and social work, there were no non-clinical tasks specified. A variety of non-clinical tasks were performed by AHAs and students, with an even spread of tasks reported across the three studies [20, 22, 27].

#### Staffing ratios

Of the 18 sources on CCRs compiled for this scoping review, three studies also reported on staffing ratios [21, 24, 25], as seen in Table 10. In all of these studies, staffing ratios were calculated by comparing the number of full-time equivalent staff to beds. The specific staffing ratios concerned a range of AHPs, including dietitians, physiotherapists, occupational therapists, speech and language therapists, social workers and psychologists, across a variety of settings.

**Table 10:** Studies which reported staffing ratios.

Study	Staffing Ratio
Bourne et al. 2019 [11]	N
Domenech et al. 1983 [13]	N
Dunal et al. 2005 [26]	N
Hall et al. 2021 [20]	N*
Hand et al. 2019 [14]	N
Hand et al. 2015 [15]	N
Hearn et al. 2017 [3]	N
Iosa et al. 2018 [27]	N
Long et al. 2020 [16]	N
Meyer and Olsen 1989 [17]	N
Milosavljevic et al. 2014 [21]	Y**
Phillips 2015 [18]	N
Pinson et al. 2023 [22]	N
Rudd et al. 2009 [24]	Y***
Shanklin et al. 1988 [12]	N
Stachowski and Kondela-Cebulski 1983 [19]	N
Stoikov et al. 2021 [23]	N
Ward and O’Riordan 2015 [25]	Y****

Note

* (Physiotherapists only) Data on FTE provided (4 FTE PT and 1 FTE AHA) however no specific staffing ratio included

** (Dietitians only) Various FTE/no. of beds ratios reported, dependent on setting (acute aged care: 0.8/56; rehab: 1/42; major regional referral centre: 6.4/465, remaining 4 sites number of beds N/A)

*** FTE staffing levels per 10 beds: Physiotherapy: 1.3, 1–2, 0.9, 0.8 (across 4 sites), Occupational therapy: 1.0, 0.9–1.3, 0.7, 0.6 (across 4 sites), Speech and language therapy: 0.3, 0.2–0.6, 0.35, 0.25 (across 4 sites), Social work: 0.4–0.7 (across 1 site), Psychology: 0 (across 1 site)

**** (Dietitians only) Various staff-bed ratios reported, ranging from 1:8 to 1:40

## Discussion

### Summary of evidence

There has been limited research exploring the direct and indirect CCRs of AHPs, with implications spanning across workforce planning, resource allocation, task delegation, and overall patient care [1, 5]. This lack of comprehensive understanding can result in inefficient workforce, resource and task allocations, which ultimately impact the quality of patient care [1, 5]. Collectively, evidence from the literature indicates considerable variability, at times within and across professions, for CCRs as well as tasks undertaken. While this may be a manifestation of local contexts informing these metrics, it poses challenges for system level planning.

### Clinical care ratios

The findings reveal AHP CCRs consist of approximately 30% of working hours allocated to activities beyond direct clinical care. This has important implications for patient contact time and overall healthcare efficiency, especially when there are considerable health workforce challenges. A study by Yen et al. [28] on nurses’ time allocation showed that nurses allocate 10% of their time to non-nursing tasks, which means a large proportion of the time is spent on patient care. One strategy for enhancing CCRs is in the expanding use of AHAs. Research by Snowdon et al. [29] and Lizarondo et al. [30] support this approach, demonstrating that AHAs, when delegated clinical responsibilities, can have a positive impact on patient care and organizational outcomes. Similarly, as revealed in Kumar et al. [31] study, students can act as change agents in healthcare, influencing knowledge, attitudes, and practices. AHAs and students together offer a multifaceted approach to improving CCRs by allowing AHPs to allocate more direct clinical time, thereby enhancing patient care and resource allocation. However, this raises an interesting conundrum. While AHAs and students can help free up direct clinical time for AHPs, their involvement may bring additional time costs through non-clinical activities such as supervision, delegation, training, and teaching. Therefore, careful consideration is required to ensure that these efforts do not merely shift responsibilities from one professional role to another (i.e. gaining time in one task only to lose it in another).

### Variability in clinical care ratios across allied health professions

This scoping review highlights the variability in CCRs among different AHPs. It establishes the differences between professions such as physiotherapy, dietetics, occupational therapy, speech pathology, and medical imaging assistants, and how their time is allocated distinctively based on the professions common clinical and non-clinical tasks. This variability is influenced by factors including the nature of each profession, patient demographics, the complexity of patient needs, and the specific care settings in which these professionals work across various health settings. For example, the demand on AHPs within an acute care hospital may differ from those in an outpatient or residential aged care setting. As observed in studies by Hall et al. [20], Domenech et al. [13], and Phillips [18], the time allocation for direct patient care varies significantly between health care settings and professions such as physiotherapy and dietetics. Furthermore, even within similar health settings such as hospital sites, both dietetics and physiotherapy professions established a wide range of CCRs ranging from 28.5% - 81% and 32.1% - 77% respectively. There is a great range and variability within CCRs across AHPs, however this could be related to inconsistencies in data across studies and the lack of universally accepted definitions of AHPs and their roles. Some studies presented ratios in terms of percentages, whilst others expressed specific time durations, subsequently adding an additional layer of diversity in how CCRs are documented.

This lack of consistency underscores the requirement for tailored, profession-specific approaches to workforce planning and resource distribution. From the data presented within this scoping review, it is evident that a one-size-fits-all strategy will not be effective in optimising quality patient care and healthcare system efficiency across AHPs. It highlights the importance of recognising that each profession’s unique roles within a multidisciplinary team is paramount in the design of effective policies and practice to ensure optimal delivery of care.

### Clinical and non-clinical tasks

Based on the findings of this scoping review, AHPs, assistants and students perform many different clinical and non-clinical tasks specific to the discipline and context in which they work. Consistency was present whereby all AHPs performed high level direct and indirect clinical tasks such as assessment, treatment, communication/meetings/medical rounds and training/education/research [3, 14, 16, 20, 27] while AHAs’ and students did lower-level indirect clinical tasks such as administration, documentation and equipment [20, 22, 27]. This potentially highlights how AHPs’ expertise could be utilised to perform more technical tasks thereby maximising their time and allowing lower-level tasks to be performed by AHAs and students.

However, there is some inconsistency across the studies as to what constitutes clinical and non-clinical work. For example, administration, documentation and training/education have been classified as both indirect clinical tasks [3, 14, 18, 20, 21] and non-clinical tasks [16–18, 20, 21, 27]. In addition, over one-quarter of the studies did not specify what clinical or non-clinical work is undertaken by these individuals. Therefore, it is difficult to draw a conclusion as to what particular tasks are being performed by AHPs, assistants and students during clinical and non-clinical time.

Moreover, while the studies considered the various tasks that constitute the different roles within each discipline, these tasks were linked to the specific institution in which the allied health worker performed their duties. To provide general guidance on what each discipline considers clinical and non-clinical work, scope of work guidelines such as the ‘*SA Health*: *Allied Health Data Set Specifications and Data Definitions’* can enable a better understanding of how clinical and non-clinical time is distributed [8]. This knowledge would also enable a clearer understanding of which particular non-clinical tasks AHAs could adopt to support the availability of clinical time for AHPs. For example, there may be scope for assistants to take on administration, which the findings suggest is a commonly performed non-clinical task undertaken by both physiotherapists and dietitians across one-third of the studies [13, 14, 18, 21, 27].

### Limitations

The limitations of this review are multiple. Despite a comprehensive search strategy, this review did not identify any literature on this topic from podiatry, social work, speech pathology, psychology, audiology, and exercise physiology. As such, findings from this review cannot be generalisable to these professions and further research is required to address this knowledge gap. As the search was limited to English language articles only, publication and language bias should be acknowledged. A consequence of this was most the publications originated from English-speaking Western countries which limit transferability of these findings to AHPs in developing countries. As AHPs continue to grow in developing countries, future research about workforce and tasks from these jurisdictions can contribute to the knowledge base on this topic.

## Conclusions

This scoping review is the first, to our knowledge, to summarise the current literature on CCRs and tasks undertaken by AHPs, AHAs and students. We found considerable variability in CCRs among different AHP disciplines, with physiotherapy and dietetics being the most represented. The heterogeneity of CCRs, and how they are measured and reported, are likely influenced by various factors, including the nature of the profession, patient demographics, and the care settings in which AHPs operate. Adding to this complexity is the ambiguity about the definition of clinical and non-clinical work, with classification of tasks, such as administration, documentation, and training/education, varying across studies. Despite these challenges, collectively the literature highlights nearly a third of time spent by AHPs are on tasks that are beyond direct clinical care. This presents an opportunity for AHAs and students to undertake such tasks, supported through supervision and delegation by AHPs, which would then free-up AHPs to undertake more direct and higher-level tasks. Future research could explore such role substitutions as well as include a wider range of professions and healthcare contexts. Future research will be pivotal in shaping future policies and practices to enhance patient care and system efficiency across all AHPs.

## Supporting information

S1 Checklist Preferred Reporting Items for Systematic reviews and Meta-Analyses extension for Scoping Reviews (PRISMA-ScR) checklist. (PDF)

S1 Appendix (DOCX)
