# Supplementary material for: Clinical care ratios for, and tasks undertaken by, allied health professionals: A scoping review of the literature
Source: PLoS One. 2024 Nov 12;19(11):e0312435. doi: 10.1371/journal.pone.0312435 (PMC11556700; doi:10.1371/journal.pone.0312435)
Supplement: S1 Appendix — (DOCX) [file pone.0312435.s002.docx]

**APPENDICES**

**APPENDIX 1: Information Sources**

| **Database:** | **Search Date:** | **Researcher Performing the Search:** |
| --- | --- | --- |
| Ovid MEDLINE | 03/10/2023 | JK and AP |
| Ovid Emcare | 03/10/2023 | JK and AP |
| Ovid Embase | 03/10/2023 | JK and AP |
| Ovid PsycINFO | 03/10/2023 | JK and AP |
| CINAHL | 03/10/2023 | JK and AP |
| The Cochrane Library | 03/10/2023 | JK and AP |
| Scopus | 03/10/2023 | JK and AP |
| PEDro | 03/10/2023 | AP and AP |
| OTseeker | 03/10/2023 | AP and AP |
| Google Scholar | 03/10/2023 | EA and FF |
| Google | 03/10/2023 | FF and EA |

**APPENDIX 2: OVID Search Syntax**

Ovid MEDLINE(R) ALL <1946 to September 29, 2023>

1 Allied Health Personnel/ 13106

2 (physiotherap* or podiatr* or "social work*" or "occupational therap*" or dieti?ian* or "allied health*" or "medical radiologist*" or psychologist* or "psychology staff*" or "speech therapist*" or "speech pathologist*" or "exercise therapist*" or "exercise physiologist*" or audiologist* or "physical therapist*").mp. [mp=title, book title, abstract, original title, name of substance word, subject heading word, floating sub-heading word, keyword heading word, organism supplementary concept word, protocol supplementary concept word, rare disease supplementary concept word, unique identifier, synonyms, population supplementary concept word, anatomy supplementary concept word] 151371

3 1 or 2 151371

4 ("clinical care ratio*" or "indirect ADJ3 direct" or "clinical ADJ3 non-clinical" or "workforce ratio*" or workload* or "staff* ratio*" or "workforce plan*" or "direct patient car*" or "indirect patient car*" or staffing).mp. [mp=title, book title, abstract, original title, name of substance word, subject heading word, floating sub-heading word, keyword heading word, organism supplementary concept word, protocol supplementary concept word, rare disease supplementary concept word, unique identifier, synonyms, population supplementary concept word, anatomy supplementary concept word] 84101

5 (acute* or inpatient* or in-patient* or outpatient* or out-patient* or hospital* or sub-acute* or subacute* or "sub acute*" or "healthcare facilit*" or "residential facilit*" or "nursing home*" or "aged car*" or rehab*).mp. [mp=title, book title, abstract, original title, name of substance word, subject heading word, floating sub-heading word, keyword heading word, organism supplementary concept word, protocol supplementary concept word, rare disease supplementary concept word, unique identifier, synonyms, population supplementary concept word, anatomy supplementary concept word] 5424352

6 3 and 4 and 5 1195

7 limit 6 to english language 1136
